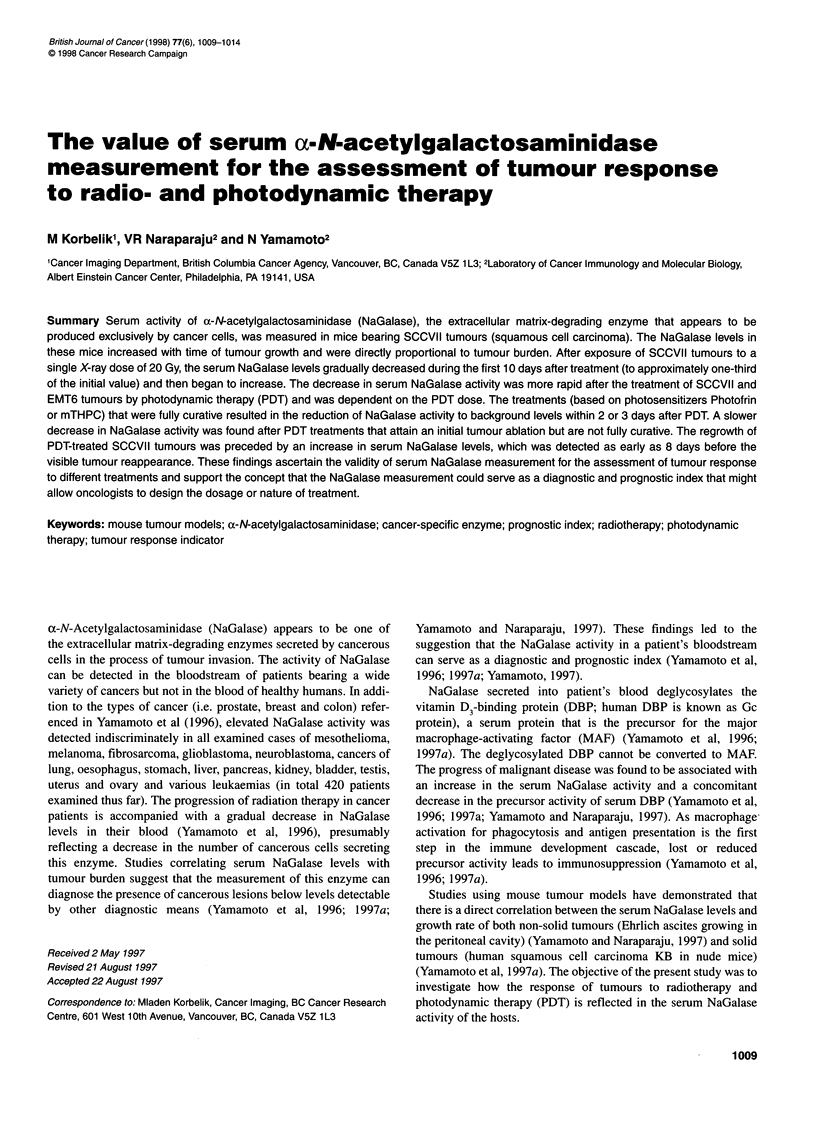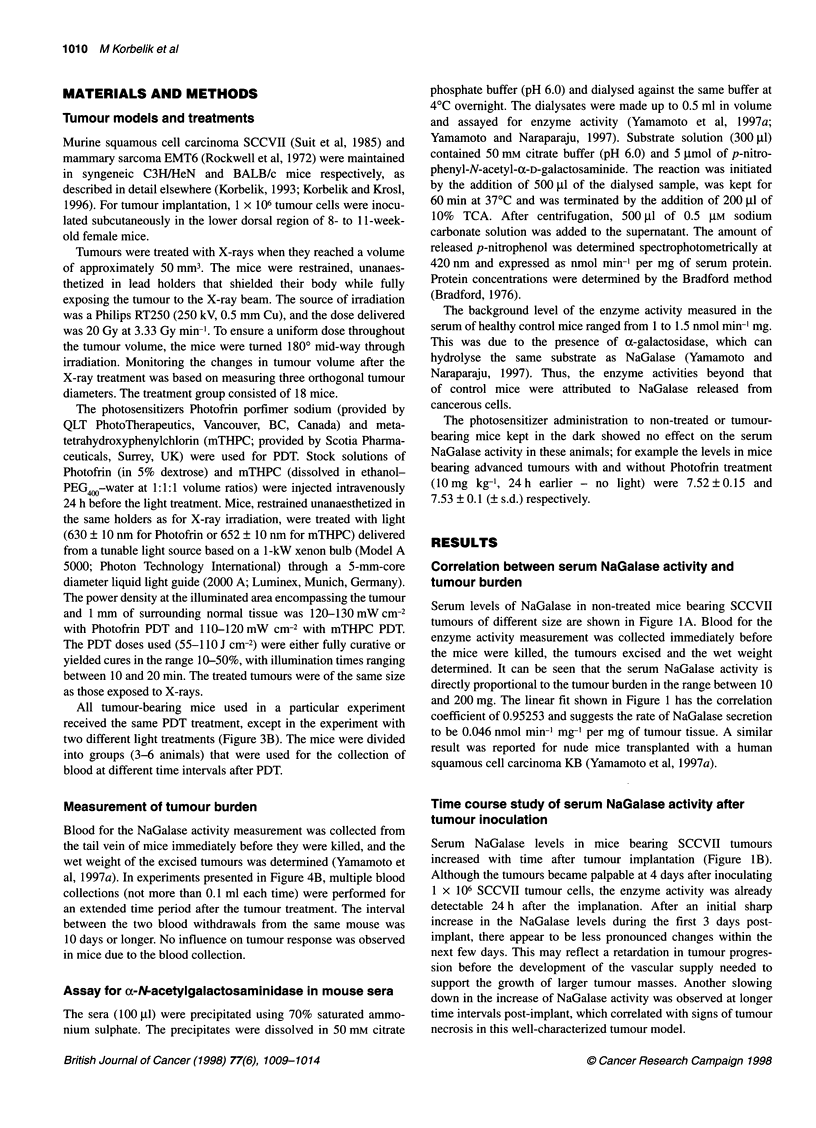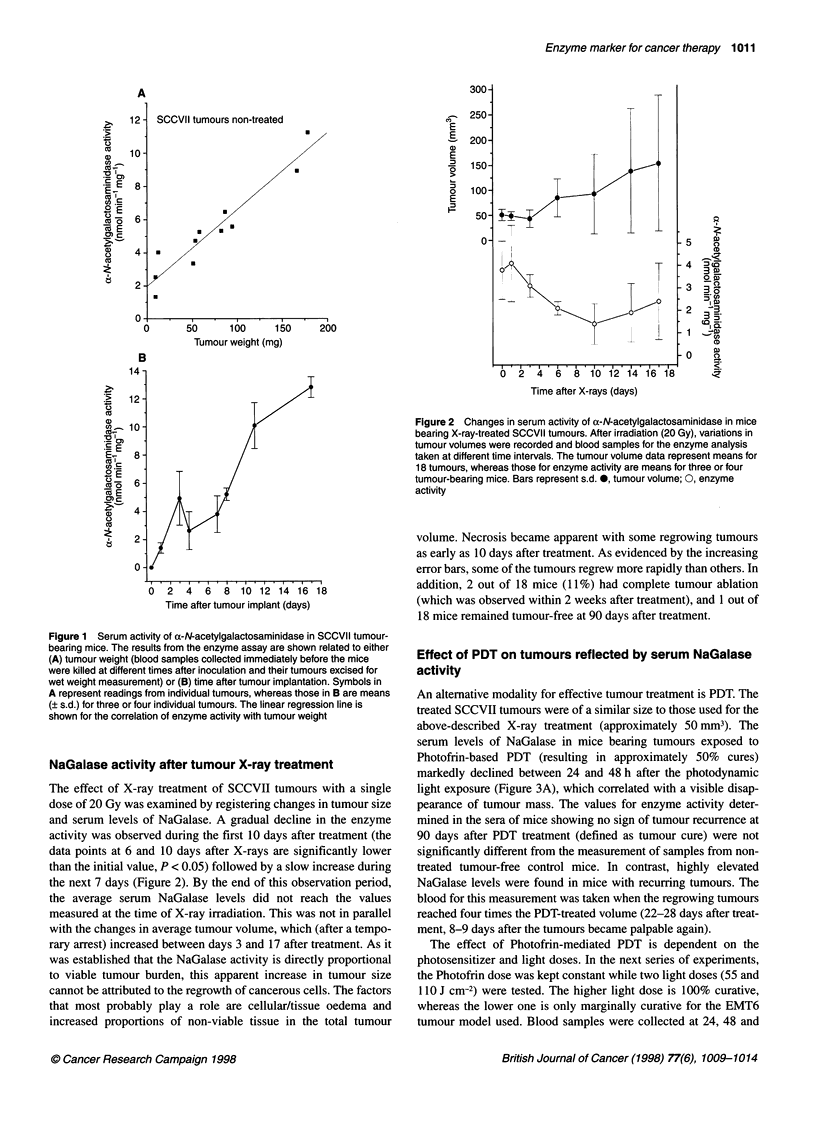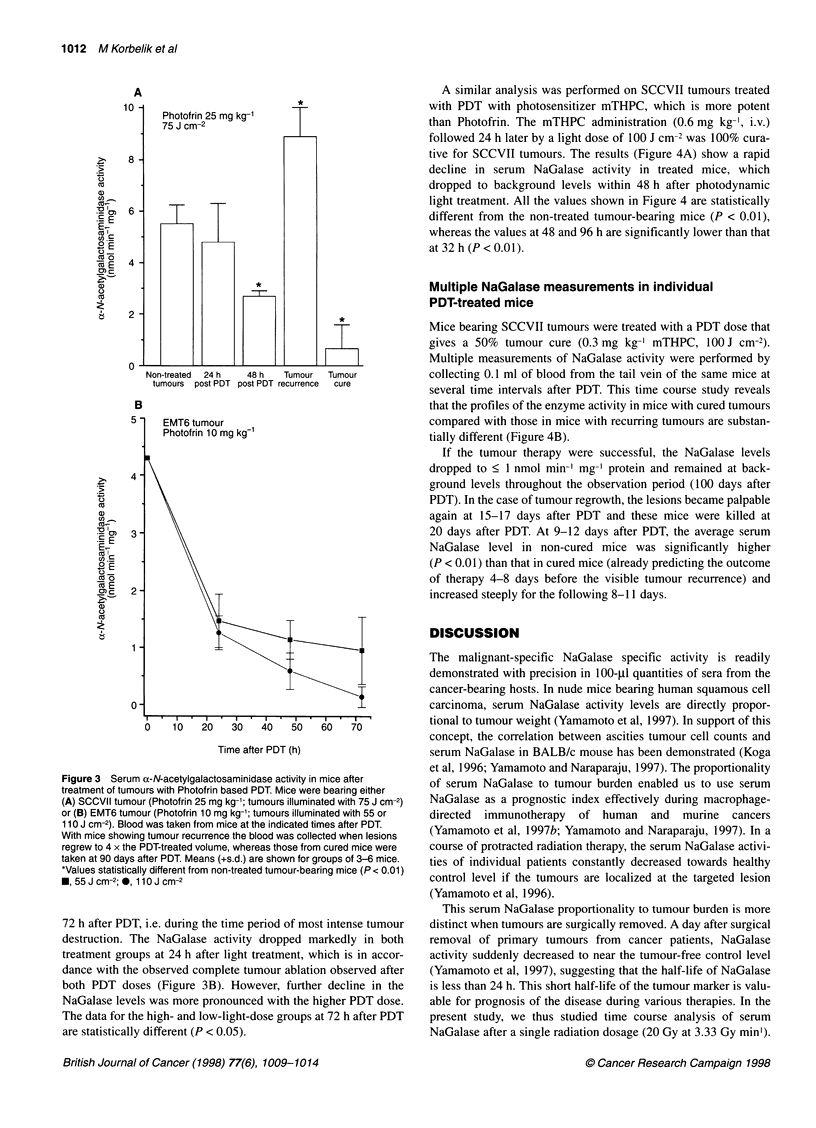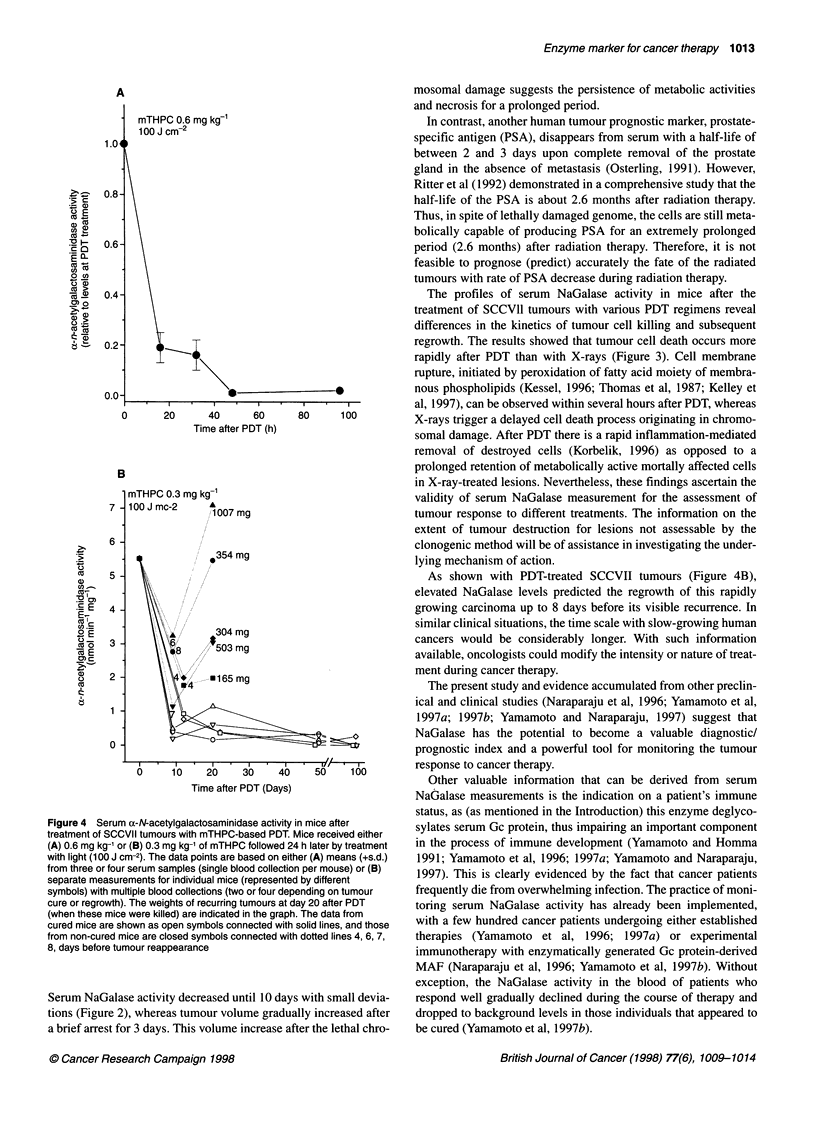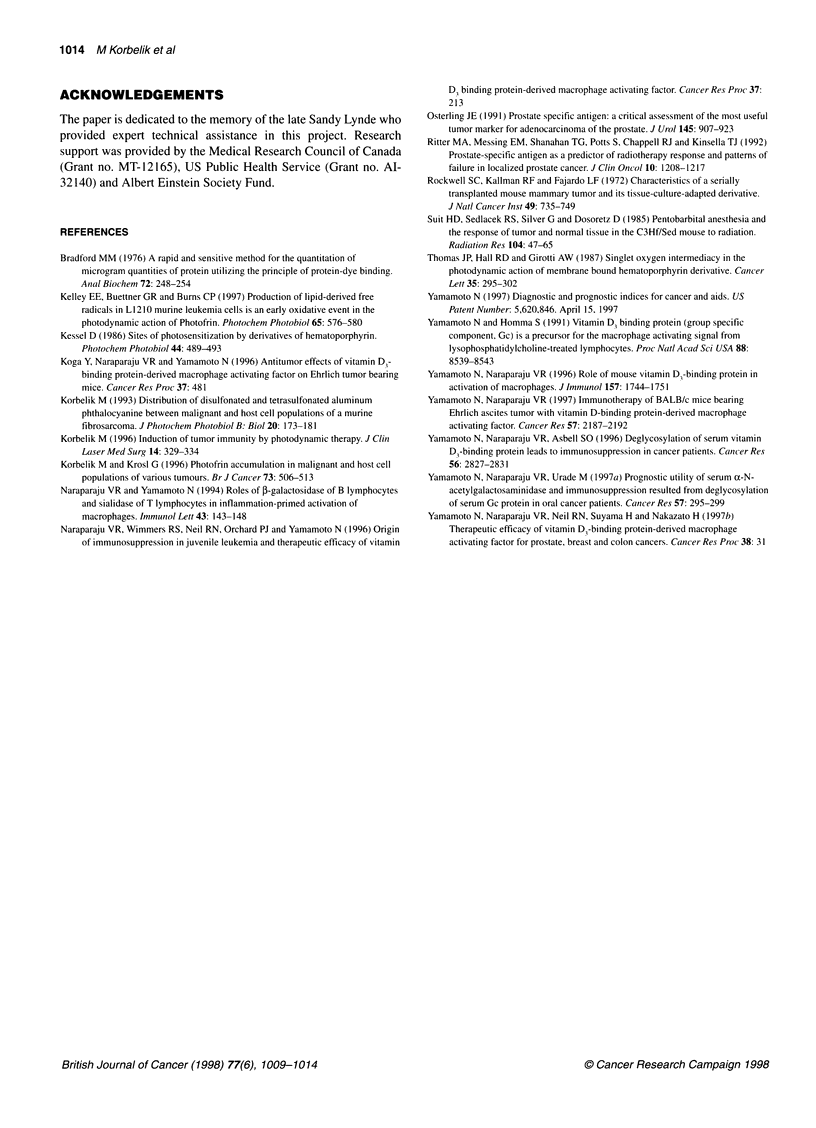# The value of serum alpha-N-acetylgalactosaminidase measurement for the assessment of tumour response to radio- and photodynamic therapy.

**DOI:** 10.1038/bjc.1998.166

**Published:** 1998-03

**Authors:** M. Korbelik, V. R. Naraparaju, N. Yamamoto

**Affiliations:** Cancer Imaging Department, British Columbia Cancer Agency, Vancouver, Canada.

## Abstract

Serum activity of alpha-N-acetylgalactosaminidase (NaGalase), the extracellular matrix-degrading enzyme that appears to be produced exclusively by cancer cells, was measured in mice bearing SCCVII tumours (squamous cell carcinoma). The NaGalase levels in these mice increased with time of tumour growth and were directly proportional to tumour burden. After exposure of SCCVII tumours to a single X-ray dose of 20 Gy, the serum NaGalase levels gradually decreased during the first 10 days after treatment (to approximately one-third of the initial value) and then began to increase. The decrease in serum NaGalase activity was more rapid after the treatment of SCCVII and EMT6 tumours by photodynamic therapy (PDT) and was dependent on the PDT dose. The treatments (based on photosensitizers Photofrin or mTHPC) that were fully curative resulted in the reduction of NaGalase activity to background levels within 2 or 3 days after PDT. A slower decrease in NaGalase activity was found after PDT treatments that attain an initial tumour ablation but are not fully curative. The regrowth of PDT-treated SCCVII tumours was preceded by an increase in serum NaGalase levels, which was detected as early as 8 days before the visible tumour reappearance. These findings ascertain the validity of serum NaGalase measurement for the assessment of tumour response to different treatments and support the concept that the NaGalase measurement could serve as a diagnostic and prognostic index that might allow oncologists to design the dosage or nature of treatment.


British Journal of Cancer (1998) 77(6), 1009–1014
© 1998 Cancer Research Campaign

### The value of serum α-*N*-acetylgalactosaminidase measurement for the assessment of tumour response to radio- and photodynamic therapy

**M Korbelik[1], VR Naraparaju[2] and N Yamamoto[2]**

[1]Cancer Imaging Department, British Columbia Cancer Agency, Vancouver, BC, Canada V5Z 1L3; [2]Laboratory of Cancer Immunology and Molecular Biology, Albert Einstein Cancer Center, Philadelphia, PA 19141, USA

**Summary** Serum activity of α-*N*-acetylgalactosaminidase (NaGalase), the extracellular matrix-degrading enzyme that appears to be produced exclusively by cancer cells, was measured in mice bearing SCCVII tumours (squamous cell carcinoma). The NaGalase levels in these mice increased with time of tumour growth and were directly proportional to tumour burden. After exposure of SCCVII tumours to a single *X*-ray dose of 20 Gy, the serum NaGalase levels gradually decreased during the first 10 days after treatment (to approximately one-third of the initial value) and then began to increase. The decrease in serum NaGalase activity was more rapid after the treatment of SCCVII and EMT6 tumours by photodynamic therapy (PDT) and was dependent on the PDT dose. The treatments (based on photosensitizers Photofrin or mTHPC) that were fully curative resulted in the reduction of NaGalase activity to background levels within 2 or 3 days after PDT. A slower decrease in NaGalase activity was found after PDT treatments that attain an initial tumour ablation but are not fully curative. The regrowth of PDT-treated SCCVII tumours was preceded by an increase in serum NaGalase levels, which was detected as early as 8 days before the visible tumour reappearance. These findings ascertain the validity of serum NaGalase measurement for the assessment of tumour response to different treatments and support the concept that the NaGalase measurement could serve as a diagnostic and prognostic index that might allow oncologists to design the dosage or nature of treatment.

**Keywords:** mouse tumour models; α-*N*-acetylgalactosaminidase; cancer-specific enzyme; prognostic index; radiotherapy; photodynamic therapy; tumour response indicator

α-*N*-Acetylgalactosaminidase (NaGalase) appears to be one of the extracellular matrix-degrading enzymes secreted by cancerous cells in the process of tumour invasion. The activity of NaGalase can be detected in the bloodstream of patients bearing a wide variety of cancers but not in the blood of healthy humans. In addition to the types of cancer (i.e. prostate, breast and colon) referenced in Yamamoto et al (1996), elevated NaGalase activity was detected indiscriminately in all examined cases of mesothelioma, melanoma, fibrosarcoma, glioblastoma, neuroblastoma, cancers of lung, oesophagus, stomach, liver, pancreas, kidney, bladder, testis, uterus and ovary and various leukaemias (in total 420 patients examined thus far). The progression of radiation therapy in cancer patients is accompanied with a gradual decrease in NaGalase levels in their blood (Yamamoto et al, 1996), presumably reflecting a decrease in the number of cancerous cells secreting this enzyme. Studies correlating serum NaGalase levels with tumour burden suggest that the measurement of this enzyme can diagnose the presence of cancerous lesions below levels detectable by other diagnostic means (Yamamoto et al, 1996; 1997*a*; Yamamoto and Naraparaju, 1997). These findings led to the suggestion that the NaGalase activity in a patient's bloodstream can serve as a diagnostic and prognostic index (Yamamoto et al, 1996; 1997*a*; Yamamoto, 1997).

*Received 2 May 1997*
*Revised 21 August 1997*
*Accepted 22 August 1997*

*Correspondence to:* Mladen Korbelik, Cancer Imaging, BC Cancer Research Centre, 601 West 10th Avenue, Vancouver, BC, Canada V5Z 1L3

NaGalase secreted into patient's blood deglycosylates the vitamin $D_3$-binding protein (DBP; human DBP is known as Gc protein), a serum protein that is the precursor for the major macrophage-activating factor (MAF) (Yamamoto et al, 1996; 1997*a*). The deglycosylated DBP cannot be converted to MAF. The progress of malignant disease was found to be associated with an increase in the serum NaGalase activity and a concomitant decrease in the precursor activity of serum DBP (Yamamoto et al, 1996; 1997a; Yamamoto and Naraparaju, 1997). As macrophage activation for phagocytosis and antigen presentation is the first step in the immune development cascade, lost or reduced precursor activity leads to immunosuppression (Yamamoto et al, 1996; 1997*a*).

Studies using mouse tumour models have demonstrated that there is a direct correlation between the serum NaGalase levels and growth rate of both non-solid tumours (Ehrlich ascites growing in the peritoneal cavity) (Yamamoto and Naraparaju, 1997) and solid tumours (human squamous cell carcinoma KB in nude mice) (Yamamoto et al, 1997*a*). The objective of the present study was to investigate how the response of tumours to radiotherapy and photodynamic therapy (PDT) is reflected in the serum NaGalase activity of the hosts.

### MATERIALS AND METHODS

### Tumour models and treatments

Murine squamous cell carcinoma SCCVII (Suit et al, 1985) and mammary sarcoma EMT6 (Rockwell et al, 1972) were maintained in syngeneic C3H/HeN and BALB/c mice respectively, as described in detail elsewhere (Korbelik, 1993; Korbelik and Krosl, 1996). For tumour implantation, $1 \times 10^6$ tumour cells were inoculated subcutaneously in the lower dorsal region of 8- to 11-week-old female mice.

Tumours were treated with X-rays when they reached a volume of approximately 50 $mm^3$. The mice were restrained, unanaesthetized in lead holders that shielded their body while fully exposing the tumour to the X-ray beam. The source of irradiation was a Philips RT250 (250 kV, 0.5 mm Cu), and the dose delivered was 20 Gy at 3.33 Gy $min^{-1}$. To ensure a uniform dose throughout the tumour volume, the mice were turned 180° mid-way through irradiation. Monitoring the changes in tumour volume after the X-ray treatment was based on measuring three orthogonal tumour diameters. The treatment group consisted of 18 mice.

The photosensitizers Photofrin porfimer sodium (provided by QLT PhotoTherapeutics, Vancouver, BC, Canada) and meta-tetrahydroxyphenylchlorin (mTHPC; provided by Scotia Pharmaceuticals, Surrey, UK) were used for PDT. Stock solutions of Photofrin (in 5% dextrose) and mTHPC (dissolved in ethanol–$PEG_{400}$–water at 1:1:1 volume ratios) were injected intravenously 24 h before the light treatment. Mice, restrained unanaesthetized in the same holders as for X-ray irradiation, were treated with light (630 ± 10 nm for Photofrin or 652 ± 10 nm for mTHPC) delivered from a tunable light source based on a 1-kW xenon bulb (Model A 5000; Photon Technology International) through a 5-mm-core diameter liquid light guide (2000 A; Luminex, Munich, Germany). The power density at the illuminated area encompassing the tumour and 1 mm of surrounding normal tissue was 120–130 mW $cm^{-2}$ with Photofrin PDT and 110–120 mW $cm^{-2}$ with mTHPC PDT. The PDT doses used (55–110 J $cm^{-2}$) were either fully curative or yielded cures in the range 10–50%, with illumination times ranging between 10 and 20 min. The treated tumours were of the same size as those exposed to X-rays.

All tumour-bearing mice used in a particular experiment received the same PDT treatment, except in the experiment with two different light treatments (Figure 3B). The mice were divided into groups (3–6 animals) that were used for the collection of blood at different time intervals after PDT.

### Measurement of tumour burden

Blood for the NaGalase activity measurement was collected from the tail vein of mice immediately before they were killed, and the wet weight of the excised tumours was determined (Yamamoto et al, 1997*a*). In experiments presented in Figure 4B, multiple blood collections (not more than 0.1 ml each time) were performed for an extended time period after the tumour treatment. The interval between the two blood withdrawals from the same mouse was 10 days or longer. No influence on tumour response was observed in mice due to the blood collection.

### Assay for α-*N*-acetylgalactosaminidase in mouse sera

The sera (100 µl) were precipitated using 70% saturated ammonium sulphate. The precipitates were dissolved in 50 mM citrate phosphate buffer (pH 6.0) and dialysed against the same buffer at 4°C overnight. The dialysates were made up to 0.5 ml in volume and assayed for enzyme activity (Yamamoto et al, 1997*a*; Yamamoto and Naraparaju, 1997). Substrate solution (300 µl) contained 50 mM citrate buffer (pH 6.0) and 5 µmol of *p*-nitrophenyl-*N*-acetyl-α-D-galactosaminide. The reaction was initiated by the addition of 500 µl of the dialysed sample, was kept for 60 min at 37°C and was terminated by the addition of 200 µl of 10% TCA. After centrifugation, 500 µl of 0.5 µM sodium carbonate solution was added to the supernatant. The amount of released *p*-nitrophenol was determined spectrophotometrically at 420 nm and expressed as nmol $min^{-1}$ per mg of serum protein. Protein concentrations were determined by the Bradford method (Bradford, 1976).

The background level of the enzyme activity measured in the serum of healthy control mice ranged from 1 to 1.5 nmol $min^{-1}$ mg. This was due to the presence of α-galactosidase, which can hydrolyse the same substrate as NaGalase (Yamamoto and Naraparaju, 1997). Thus, the enzyme activities beyond that of control mice were attributed to NaGalase released from cancerous cells.

The photosensitizer administration to non-treated or tumour-bearing mice kept in the dark showed no effect on the serum NaGalase activity in these animals; for example the levels in mice bearing advanced tumours with and without Photofrin treatment (10 mg $kg^{-1}$, 24 h earlier – no light) were 7.52 ± 0.15 and 7.53 ± 0.1 (± s.d.) respectively.

### RESULTS

### Correlation between serum NaGalase activity and tumour burden

Serum levels of NaGalase in non-treated mice bearing SCCVII tumours of different size are shown in Figure 1A. Blood for the enzyme activity measurement was collected immediately before the mice were killed, the tumours excised and the wet weight determined. It can be seen that the serum NaGalase activity is directly proportional to the tumour burden in the range between 10 and 200 mg. The linear fit shown in Figure 1 has the correlation coefficient of 0.95253 and suggests the rate of NaGalase secretion to be 0.046 nmol $min^{-1}$ $mg^{-1}$ per mg of tumour tissue. A similar result was reported for nude mice transplanted with a human squamous cell carcinoma KB (Yamamoto et al, 1997*a*).

### Time course study of serum NaGalase activity after tumour inoculation

Serum NaGalase levels in mice bearing SCCVII tumours increased with time after tumour implantation (Figure 1B). Although the tumours became palpable at 4 days after inoculating $1 \times 10^6$ SCCVII tumour cells, the enzyme activity was already detectable 24 h after the implanation. After an initial sharp increase in the NaGalase levels during the first 3 days post-implant, there appear to be less pronounced changes within the next few days. This may reflect a retardation in tumour progression before the development of the vascular supply needed to support the growth of larger tumour masses. Another slowing down in the increase of NaGalase activity was observed at longer time intervals post-implant, which correlated with signs of tumour necrosis in this well-characterized tumour model.

*© Cancer Research Campaign 1998*

**Figure 1** Serum activity of α-*N*-acetylgalactosaminidase in SCCVII tumour-bearing mice. The results from the enzyme assay are shown related to either (**A**) tumour weight (blood samples collected immediately before the mice were killed at different times after inoculation and their tumours excised for wet weight measurement) or (**B**) time after tumour implantation. Symbols in **A** represent readings from individual tumours, whereas those in **B** are means (± s.d.) for three or four individual tumours. The linear regression line is shown for the correlation of enzyme activity with tumour weight

### NaGalase activity after tumour X-ray treatment

The effect of X-ray treatment of SCCVII tumours with a single dose of 20 Gy was examined by registering changes in tumour size and serum levels of NaGalase. A gradual decline in the enzyme activity was observed during the first 10 days after treatment (the data points at 6 and 10 days after X-rays are significantly lower than the initial value, $P < 0.05$) followed by a slow increase during the next 7 days (Figure 2). By the end of this observation period, the average serum NaGalase levels did not reach the values measured at the time of X-ray irradiation. This was not in parallel with the changes in average tumour volume, which (after a temporary arrest) increased between days 3 and 17 after treatment. As it was established that the NaGalase activity is directly proportional to viable tumour burden, this apparent increase in tumour size cannot be attributed to the regrowth of cancerous cells. The factors that most probably play a role are cellular/tissue oedema and increased proportions of non-viable tissue in the total tumour volume. Necrosis became apparent with some regrowing tumours as early as 10 days after treatment. As evidenced by the increasing error bars, some of the tumours regrew more rapidly than others. In addition, 2 out of 18 mice (11%) had complete tumour ablation (which was observed within 2 weeks after treatment), and 1 out of 18 mice remained tumour-free at 90 days after treatment.

**Figure 2** Changes in serum activity of α-*N*-acetylgalactosaminidase in mice bearing X-ray-treated SCCVII tumours. After irradiation (20 Gy), variations in tumour volumes were recorded and blood samples for the enzyme analysis taken at different time intervals. The tumour volume data represent means for 18 tumours, whereas those for enzyme activity are means for three or four tumour-bearing mice. Bars represent s.d. ●, tumour volume; ○, enzyme activity

### Effect of PDT on tumours reflected by serum NaGalase activity

An alternative modality for effective tumour treatment is PDT. The treated SCCVII tumours were of a similar size to those used for the above-described X-ray treatment (approximately 50 $mm^3$). The serum levels of NaGalase in mice bearing tumours exposed to Photofrin-based PDT (resulting in approximately 50% cures) markedly declined between 24 and 48 h after the photodynamic light exposure (Figure 3A), which correlated with a visible disappearance of tumour mass. The values for enzyme activity determined in the sera of mice showing no sign of tumour recurrence at 90 days after PDT treatment (defined as tumour cure) were not significantly different from the measurement of samples from non-treated tumour-free control mice. In contrast, highly elevated NaGalase levels were found in mice with recurring tumours. The blood for this measurement was taken when the regrowing tumours reached four times the PDT-treated volume (22–28 days after treatment, 8–9 days after the tumours became palpable again).

The effect of Photofrin-mediated PDT is dependent on the photosensitizer and light doses. In the next series of experiments, the Photofrin dose was kept constant while two light doses (55 and 110 J $cm^{-2}$) were tested. The higher light dose is 100% curative, whereas the lower one is only marginally curative for the EMT6 tumour model used. Blood samples were collected at 24, 48 and

**Figure 3** Serum α-*N*-acetylgalactosaminidase activity in mice after treatment of tumours with Photofrin based PDT. Mice were bearing either (**A**) SCCVII tumour (Photofrin 25 mg $kg^{-1}$; tumours illuminated with 75 J $cm^{-2}$) or (**B**) EMT6 tumour (Photofrin 10 mg $kg^{-1}$; tumours illuminated with 55 or 110 J $cm^{-2}$). Blood was taken from mice at the indicated times after PDT. With mice showing tumour recurrence the blood was collected when lesions regrew to 4 × the PDT-treated volume, whereas those from cured mice were taken at 90 days after PDT. Means (+s.d.) are shown for groups of 3–6 mice. *Values statistically different from non-treated tumour-bearing mice ($P < 0.01$) ■, 55 J $cm^{-2}$; ●, 110 J $cm^{-2}$

72 h after PDT, i.e. during the time period of most intense tumour destruction. The NaGalase activity dropped markedly in both treatment groups at 24 h after light treatment, which is in accordance with the observed complete tumour ablation observed after both PDT doses (Figure 3B). However, further decline in the NaGalase levels was more pronounced with the higher PDT dose. The data for the high- and low-light-dose groups at 72 h after PDT are statistically different ($P < 0.05$).

A similar analysis was performed on SCCVII tumours treated with PDT with photosensitizer mTHPC, which is more potent than Photofrin. The mTHPC administration (0.6 mg $kg^{-1}$, i.v.) followed 24 h later by a light dose of 100 J $cm^{-2}$ was 100% curative for SCCVII tumours. The results (Figure 4A) show a rapid decline in serum NaGalase activity in treated mice, which dropped to background levels within 48 h after photodynamic light treatment. All the values shown in Figure 4 are statistically different from the non-treated tumour-bearing mice ($P < 0.01$), whereas the values at 48 and 96 h are significantly lower than that at 32 h ($P < 0.01$).

### Multiple NaGalase measurements in individual PDT-treated mice

Mice bearing SCCVII tumours were treated with a PDT dose that gives a 50% tumour cure (0.3 mg $kg^{-1}$ mTHPC, 100 J $cm^{-2}$). Multiple measurements of NaGalase activity were performed by collecting 0.1 ml of blood from the tail vein of the same mice at several time intervals after PDT. This time course study reveals that the profiles of the enzyme activity in mice with cured tumours compared with those in mice with recurring tumours are substantially different (Figure 4B).

If the tumour therapy were successful, the NaGalase levels dropped to ≤ 1 nmol $min^{-1}$ $mg^{-1}$ protein and remained at background levels throughout the observation period (100 days after PDT). In the case of tumour regrowth, the lesions became palpable again at 15–17 days after PDT and these mice were killed at 20 days after PDT. At 9–12 days after PDT, the average serum NaGalase level in non-cured mice was significantly higher ($P < 0.01$) than that in cured mice (already predicting the outcome of therapy 4–8 days before the visible tumour recurrence) and increased steeply for the following 8–11 days.

### DISCUSSION

The malignant-specific NaGalase specific activity is readily demonstrated with precision in 100-µl quantities of sera from the cancer-bearing hosts. In nude mice bearing human squamous cell carcinoma, serum NaGalase activity levels are directly proportional to tumour weight (Yamamoto et al, 1997). In support of this concept, the correlation between ascities tumour cell counts and serum NaGalase in BALB/c mouse has been demonstrated (Koga et al, 1996; Yamamoto and Naraparaju, 1997). The proportionality of serum NaGalase to tumour burden enabled us to use serum NaGalase as a prognostic index effectively during macrophage-directed immunotherapy of human and murine cancers (Yamamoto et al, 1997*b*; Yamamoto and Naraparaju, 1997). In a course of protracted radiation therapy, the serum NaGalase activities of individual patients constantly decreased towards healthy control level if the tumours are localized at the targeted lesion (Yamamoto et al, 1996).

This serum NaGalase proportionality to tumour burden is more distinct when tumours are surgically removed. A day after surgical removal of primary tumours from cancer patients, NaGalase activity suddenly decreased to near the tumour-free control level (Yamamoto et al, 1997), suggesting that the half-life of NaGalase is less than 24 h. This short half-life of the tumour marker is valuable for prognosis of the disease during various therapies. In the present study, we thus studied time course analysis of serum NaGalase after a single radiation dosage (20 Gy at 3.33 Gy $min^{1}$).

 © Cancer Research Campaign 1998

**Figure 4** Serum α-*N*-acetylgalactosaminidase activity in mice after treatment of SCCVII tumours with mTHPC-based PDT. Mice received either (**A**) 0.6 mg $kg^{-1}$ or (**B**) 0.3 mg $kg^{-1}$ of mTHPC followed 24 h later by treatment with light (100 J $cm^{-2}$). The data points are based on either (**A**) means (+s.d.) from three or four serum samples (single blood collection per mouse) or (**B**) separate measurements for individual mice (represented by different symbols) with multiple blood collections (two or four depending on tumour cure or regrowth). The weights of recurring tumours at day 20 after PDT (when these mice were killed) are indicated in the graph. The data from cured mice are shown as open symbols connected with solid lines, and those from non-cured mice are closed symbols connected with dotted lines 4, 6, 7, 8, days before tumour reappearance

Serum NaGalase activity decreased until 10 days with small deviations (Figure 2), whereas tumour volume gradually increased after a brief arrest for 3 days. This volume increase after the lethal chromosomal damage suggests the persistence of metabolic activities and necrosis for a prolonged period.

In contrast, another human tumour prognostic marker, prostate-specific antigen (PSA), disappears from serum with a half-life of between 2 and 3 days upon complete removal of the prostate gland in the absence of metastasis (Osterling, 1991). However, Ritter et al (1992) demonstrated in a comprehensive study that the half-life of the PSA is about 2.6 months after radiation therapy. Thus, in spite of lethally damaged genome, the cells are still metabolically capable of producing PSA for an extremely prolonged period (2.6 months) after radiation therapy. Therefore, it is not feasible to prognose (predict) accurately the fate of the radiated tumours with rate of PSA decrease during radiation therapy.

The profiles of serum NaGalase activity in mice after the treatment of SCCVll tumours with various PDT regimens reveal differences in the kinetics of tumour cell killing and subsequent regrowth. The results showed that tumour cell death occurs more rapidly after PDT than with X-rays (Figure 3). Cell membrane rupture, initiated by peroxidation of fatty acid moiety of membranous phospholipids (Kessel, 1996; Thomas et al, 1987; Kelley et al, 1997), can be observed within several hours after PDT, whereas X-rays trigger a delayed cell death process originating in chromosomal damage. After PDT there is a rapid inflammation-mediated removal of destroyed cells (Korbelik, 1996) as opposed to a prolonged retention of metabolically active mortally affected cells in X-ray-treated lesions. Nevertheless, these findings ascertain the validity of serum NaGalase measurement for the assessment of tumour response to different treatments. The information on the extent of tumour destruction for lesions not assessable by the clonogenic method will be of assistance in investigating the underlying mechanism of action.

As shown with PDT-treated SCCVII tumours (Figure 4B), elevated NaGalase levels predicted the regrowth of this rapidly growing carcinoma up to 8 days before its visible recurrence. In similar clinical situations, the time scale with slow-growing human cancers would be considerably longer. With such information available, oncologists could modify the intensity or nature of treatment during cancer therapy.

The present study and evidence accumulated from other preclinical and clinical studies (Naraparaju et al, 1996; Yamamoto et al, 1997*a*; 1997*b*; Yamamoto and Naraparaju, 1997) suggest that NaGalase has the potential to become a valuable diagnostic/prognostic index and a powerful tool for monitoring the tumour response to cancer therapy.

Other valuable information that can be derived from serum NaGalase measurements is the indication on a patient's immune status, as (as mentioned in the Introduction) this enzyme deglycosylates serum Gc protein, thus impairing an important component in the process of immune development (Yamamoto and Homma 1991; Yamamoto et al, 1996; 1997*a*; Yamamoto and Naraparaju, 1997). This is clearly evidenced by the fact that cancer patients frequently die from overwhelming infection. The practice of monitoring serum NaGalase activity has already been implemented, with a few hundred cancer patients undergoing either established therapies (Yamamoto et al, 1996; 1997*a*) or experimental immunotherapy with enzymatically generated Gc protein-derived MAF (Naraparaju et al, 1996; Yamamoto et al, 1997*b*). Without exception, the NaGalase activity in the blood of patients who respond well gradually declined during the course of therapy and dropped to background levels in those individuals that appeared to be cured (Yamamoto et al, 1997*b*).

© Cancer Research Campaign 1998

### ACKNOWLEDGEMENTS

The paper is dedicated to the memory of the late Sandy Lynde who provided expert technical assistance in this project. Research support was provided by the Medical Research Council of Canada (Grant no. MT-12165), US Public Health Service (Grant no. AI-32140) and Albert Einstein Society Fund.

### REFERENCES

Bradford MM (1976) A rapid and sensitive method for the quantitation of microgram quantities of protein utilizing the principle of protein-dye binding. *Anal Biochem* **72**: 248–254

Kelley EE, Buettner GR and Burns CP (1997) Production of lipid-derived free radicals in L1210 murine leukemia cells is an early oxidative event in the photodynamic action of Photofrin. *Photochem Photobiol* **65**: 576–580

Kessel D (1986) Sites of photosensitization by derivatives of hematoporphyrin. *Photochem Photobiol* **44**: 489–493

Koga Y, Naraparaju VR and Yamamoto N (1996) Antitumor effects of vitamin $D_3$-binding protein-derived macrophage activating factor on Ehrlich tumor bearing mice. *Cancer Res Proc* **37**: 481

Korbelik M (1993) Distribution of disulfonated and tetrasulfonated aluminum phthalocyanine between malignant and host cell populations of a murine fibrosarcoma. *J Photochem Photobiol B: Biol* **20**: 173–181

Korbelik M (1996) Induction of tumor immunity by photodynamic therapy. *J Clin Laser Med Surg* **14**: 329–334

Korbelik M and Krosl G (1996) Photofrin accumulation in malignant and host cell populations of various tumours. *Br J Cancer* **73**: 506–513

Naraparaju VR and Yamamoto N (1994) Roles of β-galactosidase of B lymphocytes and sialidase of T lymphocytes in inflammation-primed activation of macrophages. *Immunol Lett* **43**: 143–148

Naraparaju VR, Wimmers RS, Neil RN, Orchard PJ and Yamamoto N (1996) Origin of immunosuppression in juvenile leukemia and therapeutic efficacy of vitamin $D_3$ binding protein-derived macrophage activating factor. *Cancer Res Proc* **37**: 213

Osterling JE (1991) Prostate specific antigen: a critical assessment of the most useful tumor marker for adenocarcinoma of the prostate. *J Urol* **145**: 907–923

Ritter MA, Messing EM, Shanahan TG, Potts S, Chappell RJ and Kinsella TJ (1992) Prostate-specific antigen as a predictor of radiotherapy response and patterns of failure in localized prostate cancer. *J Clin Oncol* **10**: 1208–1217

Rockwell SC, Kallman RF and Fajardo LF (1972) Characteristics of a serially transplanted mouse mammary tumor and its tissue-culture-adapted derivative. *J Natl Cancer Inst* **49**: 735–749

Suit HD, Sedlacek RS, Silver G and Dosoretz D (1985) Pentobarbital anesthesia and the response of tumor and normal tissue in the C3Hf/Sed mouse to radiation. *Radiation Res* **104**: 47–65

Thomas JP, Hall RD and Girotti AW (1987) Singlet oxygen intermediacy in the photodynamic action of membrane bound hematoporphyrin derivative. *Cancer Lett* **35**: 295–302

Yamamoto N (1997) Diagnostic and prognostic indices for cancer and aids. *US Patent Number*: 5,620,846. April 15, 1997

Yamamoto N and Homma S (1991) Vitamin $D_3$ binding protein (group specific component, Gc) is a precursor for the macrophage activating signal from lysophosphatidylcholine-treated lymphocytes. *Proc Natl Acad Sci USA* **88**: 8539–8543

Yamamoto N, Naraparaju VR (1996) Role of mouse vitamin $D_3$-binding protein in activation of macrophages. *J Immunol* **157**: 1744–1751

Yamamoto N, Naraparaju VR (1997) Immunotherapy of BALB/c mice bearing Ehrlich ascites tumor with vitamin D-binding protein-derived macrophage activating factor. *Cancer Res* **57**: 2187–2192

Yamamoto N, Naraparaju VR, Asbell SO (1996) Deglycosylation of serum vitamin $D_3$-binding protein leads to immunosuppression in cancer patients. *Cancer Res* **56**: 2827–2831

Yamamoto N, Naraparaju VR, Urade M (1997*a*) Prognostic utility of serum α-N-acetylgalactosaminidase and immunosuppression resulted from deglycosylation of serum Gc protein in oral cancer patients. *Cancer Res* **57**: 295–299

Yamamoto N, Naraparaju VR, Neil RN, Suyama H and Nakazato H (1997*b*) Therapeutic efficacy of vitamin $D_3$-binding protein-derived macrophage activating factor for prostate, breast and colon cancers. *Cancer Res Proc* **38**: 31

© Cancer Research Campaign 1998